# A Novel Approach to Dining Bowl Reconstruction for Image-Based Food Volume Estimation

**DOI:** 10.3390/s22041493

**Published:** 2022-02-15

**Authors:** Wenyan Jia, Yiqiu Ren, Boyang Li, Britney Beatrice, Jingda Que, Shunxin Cao, Zekun Wu, Zhi-Hong Mao, Benny Lo, Alex K. Anderson, Gary Frost, Megan A. McCrory, Edward Sazonov, Matilda Steiner-Asiedu, Tom Baranowski, Lora E. Burke, Mingui Sun

**Affiliations:** 1 Department of Electrical and Computer Engineering, University of Pittsburgh, Pittsburgh, PA 15260, USA; wej6@pitt.edu (W.J.); yir4@pitt.edu (Y.R.); bol33@pitt.edu (B.L.); Jingda.Que@hhgrace.com (J.Q.); ivy.csx@gmail.com (S.C.); zew19@pitt.edu (Z.W.); zhm4@pitt.edu (Z.-H.M.); 2 School of Health and Rehabilitation Sciences, University of Pittsburgh, Pittsburgh, PA 15260, USA; bbeatrice@pitt.edu; 3 Department of Bioengineering, University of Pittsburgh, Pittsburgh, PA 15260, USA; 4 Hamlyn Centre, Imperial College London, London SW7 2AZ, UK; benny.lo@imperial.ac.uk; 5 Department of Nutritional Sciences, University of Georgia, Athens, GA 30602, USA; fianko@uga.edu; 6 Section for Nutrition Research, Department of Metabolism, Digestion and Reproduction, Imperial College London, London SW7 2AZ, UK; g.frost@imperial.ac.uk; 7 Department of Health Sciences, Boston University, Boston, MA 02210, USA; mamccr@bu.edu; 8 Department of Electrical and Computer Engineering, University of Alabama, Tuscaloosa, AL 35487, USA; esazonov@eng.ua.edu; 9 Department of Nutrition and Food Science, University of Ghana, Legon Boundary, Accra LG 1181, Ghana; tillysteiner@gmail.com; 10 USDA/ARS Children’s Nutrition Research Center, Department of Pediatrics, Baylor College of Medicine, Houston, TX 77030, USA; tom.baranowski@bcm.edu; 11 School of Nursing, University of Pittsburgh, Pittsburgh, PA 15260, USA; lbu100@pitt.edu; 12 Department of Neurosurgery, University of Pittsburgh, Pittsburgh, PA 15260, USA

**Keywords:** 3D reconstruction, food volume estimation, image-based dietary assessment, round bowl

## Abstract

Knowing the amounts of energy and nutrients in an individual’s diet is important for maintaining health and preventing chronic diseases. As electronic and AI technologies advance rapidly, dietary assessment can now be performed using food images obtained from a smartphone or a wearable device. One of the challenges in this approach is to computationally measure the volume of food in a bowl from an image. This problem has not been studied systematically despite the bowl being the most utilized food container in many parts of the world, especially in Asia and Africa. In this paper, we present a new method to measure the size and shape of a bowl by adhering a paper ruler centrally across the bottom and sides of the bowl and then taking an image. When observed from the image, the distortions in the width of the paper ruler and the spacings between ruler markers completely encode the size and shape of the bowl. A computational algorithm is developed to reconstruct the three-dimensional bowl interior using the observed distortions. Our experiments using nine bowls, colored liquids, and amorphous foods demonstrate high accuracy of our method for food volume estimation involving round bowls as containers. A total of 228 images of amorphous foods were also used in a comparative experiment between our algorithm and an independent human estimator. The results showed that our algorithm overperformed the human estimator who utilized different types of reference information and two estimation methods, including direct volume estimation and indirect estimation through the fullness of the bowl.

## 1. Introduction

Image-based dietary assessment using a wearable camera (e.g., eButton) or a smartphone has been increasingly adopted in the study of nutrition and health [1,2,3,4,5,6,7,8,9,10]. To monitor the intake of energy and nutrients accurately, each food in the image must be identified and its volume estimated. Although food recognition has been extensively studied using deep learning techniques [11,12,13,14,15,16,17], estimating food volume from images remains a challenging problem [9,10,11,18]. Several sensor-based approaches have been reported [18,19,20,21,22,23,24,25,26,27,28,29,30,31,32,33]. A special imaging sensor called a depth sensor has been used to produce depth on a per-pixel basis from which food volume can be estimated [22,23,24,25,26]. Another effective approach uses a pair of stereo cameras separated by a distance. Food volume is estimated based on stereoscopic vision, a mechanism similar to depth perception by human eyes [27,28,29,33]. The structural light method is also an effective approach to capture 3D information. This method uses an optical scanning device to produce a light grid. When projected onto a food surface, this grid appears to be distorted. The 3D surface is then reconstructed from the observed distortion [30,31]. Although these sensor-based approaches are effective, the depth and structured light sensors are costly. Their sizes, weights, and power consumptions cause additional concerns when they are utilized within a wearable device. The stereo camera approach is less costly, but it suffers from the power consumption problem. In addition, it requires a sufficient separation between the two cameras, which elongates the wearable device, affecting its wearability. Because of these issues, current food volume estimation methods mostly use ordinary images (i.e., RGB images) in two dimensions (2D).

In general, it is difficult, if not impossible, to reconstruct a 3D surface of an amorphous food from a single 2D image or several 2D images taken from closely spaced viewpoints because (1) the whole 3D object is usually not fully observable from the image(s) and (2) a scale factor relating the physical space and the image space is missing [34]. Due to these limitations, the volume of food in a 2D image can only be estimated roughly and an estimation error, sometimes considerably large, must be tolerated [35,36]. Several estimation methods have been reported [10,17,18,35,37,38,39,40,41,42,43,44,45,46,47,48,49]. A set of computer-generated three-dimensional wireframes is used to conform to food surfaces and compute food volumes [37,38,39,40,41,42]. Although simple and effective, this method must be manually performed. Videos or multiple images from different views are also used to reconstruct the shape of the food and estimate its volume, but a fiducial marker is still required to provide the scale information [50,51,52]. Recently, AI-based methods are proposed that estimate depths of food in an image or food calories directly using deep networks [47,48,49]. However, their inaccuracy and depth/calorie uncertainty are major drawbacks. Recently, deep networks have been utilized to reconstruct 3D objects from a single image [53,54,55], but the networks need to be trained by large 3D datasets, such as synthetic datasets [55,56] or 3D scanner produced ones [57], which provide complete surface points of objects in 3D space. Due to the complexity of food in geometric attributes and shapes, there is no such food dataset currently available. A novel network, called the hungry network, has been proposed to reconstruct both the food and plate from a single image for estimating food volume [57]. A 3D dataset consisting of 240 models of foods and 38 models of plates (both in 3D) are used to train the hungry network. Since the size of this dataset is relatively small for deep learning, it is hard for the hungry network to reconstruct food or a food container that the network has never seen. In addition, a scale factor in the image is still necessary to obtain the actual food volume besides the 3D shape. To obtain the missing scale factor in an image, an object with known physical dimensions is often used as a size reference, such as a checkerboard card [6], a coin [51], a standardized cube [44,45,46], or a food serving object with known size (e.g., a circular plate [37] or a pair of chopsticks [43]).

Because food is usually contained on/in a plate/bowl, it would be convenient and advantageous to use the food container as the scale reference. In this method, the plate/bowl is pre-measured. When the container reappears in the images acquired during a dietary study, the pre-measured information is used to determine the volume of food or beverage inside the container. While measuring the diameter of a shallow plate is usually sufficient to provide a reference [58], measuring the shape and size of a bowl in the home environment represents a significant challenge to both field researchers and research participants. There is a strong need to meet this challenge because bowls are primary food containers in many parts of the world, especially in Asia and Africa [59]. In addition, meeting the bowl measurement challenge reduces the previously mentioned food volume estimation error and its uncertainty. The reason is simple: for amorphous food within a bowl, a major part of the food is confined by the known shape of the bowl except for the free-standing part on the top.

**Figure 1:**
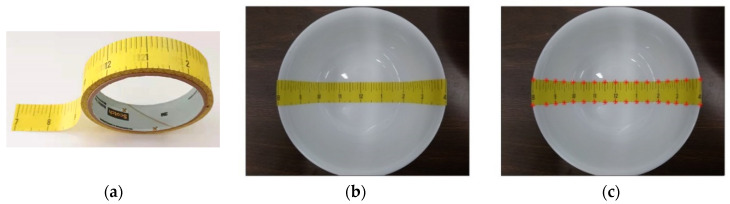
(**a**) A roll of the adhesive paper ruler as a tool for bowl measurement, (**b**) a bowl taped with an adhesive paper ruler centrally across the bottom and sides of the bowl, and (**c**) selected landmark points for computation (red asterisks).

In this work, we proposed a convenient way to pre-measure the size and shape of a bowl using an adhesive paper strip printed with ruler markers as the measurement tool (Figure 1a). This paper ruler is pasted centrally across the bottom and sides of the bowl and then a photograph is taken from the top using a smartphone or a camera (Figure 1b). Because the equally spaced markers (in a chosen physical unit, e.g., inch) on the tape become uneven and the strip width varies when observed from the image, these markers and variations provide unique information about the shape and the size of the bowl. We extract and process the information to reconstruct the interior shape of the bowl in 3D computationally. After the reconstruction, for each image containing the reconstructed bowl, the orientation and the location of the bowl relative to the camera are first estimated (details described in Section 2.2). Next, the reconstructed bowl with pre-marked volume levels is projected onto the bowl in the image based on the calculated location and orientation of the bowl. Finally, the food volume is estimated from the observed level of food within the bowl.

## 2. Methods

### 2.1. 3D Reconstruction of the Bowl

When observed from the top (Figure 1b), both the width of the yellow paper ruler and the spacings between the black markers vary in different parts of the bowl despite the ruler having a constant width and marker spacing. We use these observed variations to reconstruct the 3D shape of the bowl since these variations “encode” both the shape and the size of the bowl. Moreover, the paper ruler has some features favorable to our application: its color is fixed, and its surface is anti-reflective. Because of these properties, which are independent of the bowl, the performance of our method is stable and not affected by the material properties such as the reflectivity, decorative pattern, and color of the bowl.

Our method consists of four components as described below.

#### 2.1.1. Landmark Labeling

We selectively label the markers (shown as red asterisks in Figure 1c) to form two sets of landmark points in the image, one set along the top border of the paper ruler and the other along the bottom. For example, the labeled interval between adjacent markers in Figure 1b is 0.5 inches (which is the curve length of the tape on the bowl surface) except at the two endpoints. In this study, landmark labeling is performed manually. For a large amount of labeling, image processing algorithms could be developed to accelerate the process.

Next, we perform a coordinate transformation to convert the image coordinates of each point ${\left[x,y\right]}^{T}$ in the image (in pixel) to the coordinates ${\left[x^{\prime },y^{\prime }\right]}^{T}$ in the image plane (in a metric unit, e.g., millimeter) within the camera (the digital image is obtained from a microsensor array located in the image plane) [34]:

(1) $$\left[\begin{array}{c} x^{\prime }\\ y^{\prime } \end{array}\right]=\left[\begin{array}{cc} s_{x} & 0\\ 0 & s_{y} \end{array}\right]\left[\begin{array}{c} x\\ y \end{array}\right]+\left[\begin{array}{c} o_{x}\\ o_{y} \end{array}\right]$$

where $s_{x}\;\mathrm{and}\;s_{y}\;$ define the sizes in pixels (in the real-world physical units) of the microsensor array along x and y directions, respectively, and $o_{x}\;\mathrm{and}\;o_{y}\;$ are the coordinates (in pixels) of the principal point relative to the center of the image plane. Note that $s_{x},s_{y},\;o_{x},\;\mathrm{and}\;o_{y}$ are the intrinsic parameters of the camera. In the following description, the landmarks are in the image plane and their coordinates are in the real-world physical units. We next use the pin-hole model to approximate the projection of 3D points in the physical space to the image plane.

#### 2.1.2. Landmark Pairing and Camera Modeling

**Figure 2:**
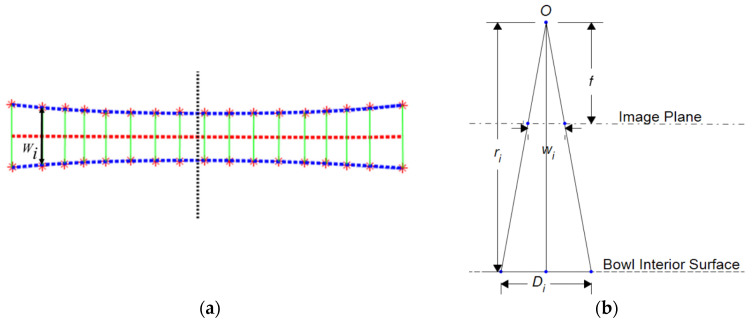
(**a**) Extracted landmarks on the image plane. The dashed vertical black line represents the central vertical line of the image. (**b**) Pinhole camera model, where *O* and *f* are the optical center and the focal length of the camera, respectively, $W_{i}\;$ represents the width (in pixels) of the observed ruler at the *i*th landmark location, $D_{i}$ is the physical distance on the ruler corresponding to the distance between the *i*th pair of landmarks in the image, and $r_{i}$ is the distance between optical center *O* and the center of the *i*th pair of landmarks on the bowl surface.

Since the detected landmark points involve certain errors, we use the least-square fitting to obtain two smooth curves (blue dash lines in Figure 2a). A 5th order polynomial is utilized in this fitting. Then, the midline between the upper and lower polynomial curves is determined by connecting midpoints of the two curves (the red dash line in Figure 2a). Finally, lines connecting corresponding pairs of landmarks are drawn (green bars). The length $W_{i}\;$ of the line represents the width of the observed ruler at the *i*th landmark location for *i* = 1, 2, ⋯, *I*. Based on the standard pinhole camera model [34], the distance $r_{i}$ between the optical center *O* and the center of the *i*th bar on the bowl surface in the 3D physical space (Figure 2b) can be estimated by

(2) $$r_{i}=fD_{i}/W_{i}$$

where *f* is the focal length of the camera (another intrinsic parameter of the camera) and $D_{i}$ is the physical distance on the ruler corresponding to the distance between the *i*th pair of landmarks in the image. Both *f* and $D_{i}\;$. are shown in Figure 2b. Equation (2) is used as the initial condition in an optimization process (to be described). Strictly speaking, $D_{i}$ cannot be determined without knowing the shape of the bowl. However, the local curvature of the bowl across the width of the ruler is small so that the curve length, which equals the known width of the ruler, can approximate $D_{i}\;$ closely.

#### 2.1.3. Formulation of Parameter Estimation

**Figure 3:**
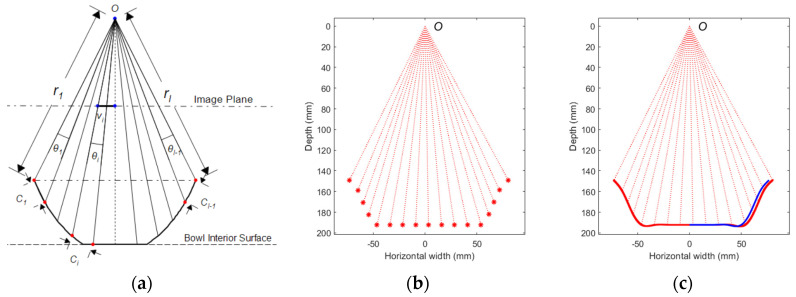
(**a**) Pinhole camera model for the reconstruction of the cross-section curve, (**b**) reconstructed intersecting points of rays between optical center *O* and bowl interior surface, and (**c**) reconstructed cross-section curves after interpolation (red), shift, rotation, and averaging (blue).

Again, we use the pinhole model as shown in Figure 3a. A set of rays initiates from the optical center of the camera through the detected landmarks intersecting with the cross-section of the bowl. Since the location of each landmark in the image is known, the angle $\;\theta_{i}$ between two adjacent rays can be calculated as:

(3) $$\theta_{i}=\mathrm{arctg}\left(V_{i}/f\right)-\mathrm{arctg}\left(V_{i+1}/f\right)$$

where $\;V_{i}$ is the signed distance between the center of the image plane and the center of the *i*th landmark (indicated in Figure 3a). This signed distance takes a positive value if the landmark locates in the left half image plane and a negative value otherwise. Let ${\hat{C}}_{i}\;$ denote an estimate of $C_{i},\;$ which is the curve length of the tape (e.g., 0.5 inches in Figure 1b except at tape ends), and the law of cosines yields

(4) $$\;{\hat{C}}_{i}=\sqrt{r_{i}{}^{2}+r_{i+1}{}^{2}-2r_{i}r_{i+1}\cos\theta_{i}}$$

where $r_{i}\;$ is the *i*th ray length. Equation (4) is an approximation because $C_{i}\;$ is a curve length rather than a distance. We form the following objective function *J*:

(5) $$J=\;\sum_{i=1}^{I-1}{\left({\hat{C}}_{i}-C_{i}\right)}^{2}=\sum_{i=1}^{I-1}{\left(\sqrt{r_{i}{}^{2}+r_{i+1}{}^{2}-2r_{i}r_{i+1}\cos\theta_{i}}-C_{i}\right)}^{2}$$

Our goal is to find the optimal values of $r_{i}$ for *i* = 1, 2, ⋯, *I* with known values of $\theta_{i}$ and $C_{i}\;$ by minimizing *J*. Considering that the bottom of the bowl is always flat, we further constrain $r_{i}\;$ by

(6) $$r_{k}\cos\left(\mathrm{arctg}\left(V_{k}/f\right)\right)=r_{k+1}\cos\left(\mathrm{arctg}\left(V_{k+1}/f\right)\right),\;\;\;\;\;k\;\in ß$$

where ß is the region of the flat bottom. ß can be specified visually from the image. With the constraint imposed, the optimization process is implemented using a nonlinear programming algorithm presented in [60]. In this optimization process, Equation (2) is used as the initial condition.

#### 2.1.4. Reconstruction of the Cross-Section Curve

**Figure 4:**
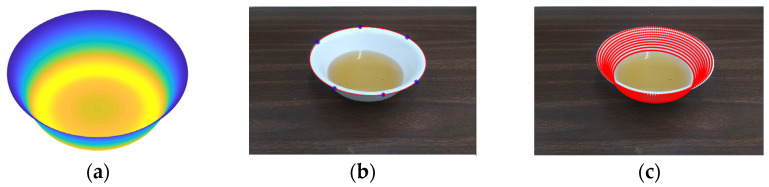
(**a**) Computationally reconstructed interior surface of the bowl. (**b**) An image of the same bowl containing one cup (237 mL) of red tea. Six points on the rim of the bowl are manually specified to fit an ellipse. (**c**) Virtual volumetric levels (red ellipses) are superimposed in the image, where each level (upwards) represents a 50 mL increment.

The optimization procedure yields the intersecting points of rays on the bowl’s interior surface, shown as the red asterisks in Figure 3b. These points are then cubic-spline interpolated to obtain a smooth curve (red solid line in Figure 3c). Since the optimization process may not guarantee the curve to be symmetric while a round bowl must be, the following post-processing is utilized: First, we shift the curve such that its center is aligned with the optical center. Then, we rotate the curve if the heights of the left and right endpoints are not the same. Next, the left and right sides of the bowl are averaged resulting in the blue curve in Figure 3c, which is the reconstructed cross-section curve of the bowl. Finally, the blue curve is rotated 360° along the vertical axis to form the entire bowl interior. Figure 4a shows an example of a reconstructed bowl. After reconstruction, all the parameters of the bowl (such as diameter, depth, and volume) can be calculated.

### 2.2. Food Volume Estimation

To estimate the volume of food when the pre-measured bowl reappears in the image acquired during a dietary study, the location and orientation of this bowl are first estimated. A useful feature of the bowl is its circular rim which appears as an ellipse in the image (Figure 4b). This ellipse can be extracted semi-manually by specifying five or more points (we usually use between six and eight) on the observed elliptic rim. Then, the selected points are fitted by an ellipse using a least-square fitting [37,58]. The orientation of the bowl can be obtained using the detected bowl rim in the image and the location of the bowl can be determined when the diameter of the bowl is known (here we use the diameter of the reconstructed bowl) [58,61]. Once the location and orientation are obtained, we superimpose the observed and reconstructed bowls by a 2D projection of the 3D bowl (shown as dashed red ellipses in Figure 4c) [34]. A sequence of levels is used to represent increments of the volumes in the projected bowl. For example, each level in Figure 4c represents a 50 mL increment. Finally, the volume of food or beverage is estimated by counting (for liquids) or estimating (for most solid or semi-solid foods) the number of levels, to be discussed in more detail below.

**Figure 5:**
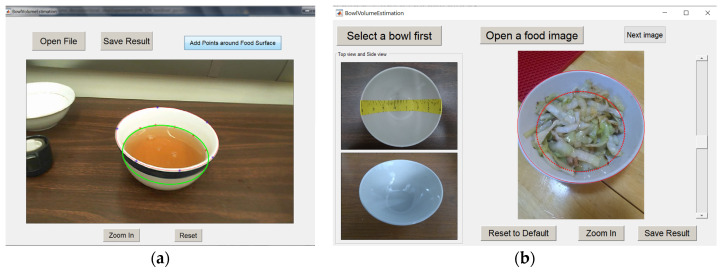
The user interface for estimating food with a flat surface (**a**) and without a flat surface (**b**).

We have developed a software interface to facilitate the volume estimation process. For liquids (e.g., drinks, soup, or porridge) with a flat top surface, we first interactively select at least five points to represent the elliptic level surface of the food. Then, all the projected ellipses are searched to find the closest match (using finer levels when necessary) shown as the green ellipse in Figure 5a. The best match provides the estimated volume.

For amorphous foods without a flat surface, we utilize a computer-assisted procedure. Our software interface (shown in Figure 5b) provides a sliding bar (right side of Figure 5b). When this bar is slid, the level line (red ellipse in the middle panel of Figure 5b) moves up or down, providing an effective visual reference to facilitate the mental flattening of the food surface and match the result with the level line. Again, the best match provides the estimated volume.

### 2.3. Alternative Method for Liquid Volume Estimation

**Figure 6:**
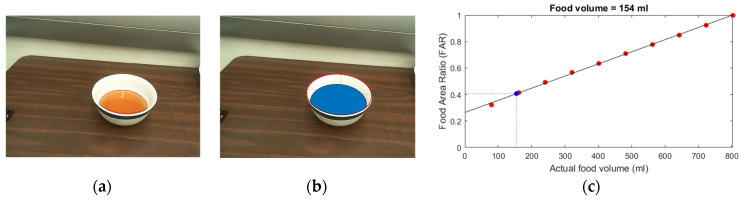
(**a**) The actual image and the segmented liquid area, (**b**) simulated liquid volume in a bowl, and (**c**) relationship between the FAR and the liquid volume. The blue asterisk in (**c**) represents the FAR of the liquid in (**b**).

In practice, manual determination of the boundary between the liquid and the interior surface of the bowl (Figure 5a) involves a certain error. Since the area at the top surface of the liquid is the largest among all parallel surfaces within the bowl, a small error in liquid level estimation results in a larger error in volume estimation. To reduce the volumetric error and facilitate automation of the estimation process, we present an alternative method by estimating the liquid area rather than the liquid level using the following steps: (1) For a given image, as shown in Figure 4b or Figure 5a, we first extract the boundary (i.e., the rim) of the bowl (an ellipse). The number of pixels within the bowl rim is computed. (2) We segment the region of the visible liquid (e.g., the light brown region in Figure 6a) and count the number of pixels in this region. (3) We compute the Food Area Ratio (FAR) defined as the ratio between the number of pixels of the visible liquid and the number of pixels within the bowl rim. (4) Using the same method, we calculate the FAR values corresponding to equal increments in volumes of a virtual (simulated) liquid in the same bowl (e.g., blue regions in Figure 6b). The relationship between the FAR values and the volume of the simulated liquid is shown as the red asterisks in Figure 6c. A regression line is computed to approximate the relationship between the FAR and food volume based on the calculated points in Figure 6c. (5) Finally, the FAR of the liquid (light brown liquid in Figure 6a) is located on the vertical axis (Figure 6c) and the corresponding volumetric value is determined from the regression line (dashed lines and blue asterisk in Figure 6c).

## 3. Experiments

### 3.1. Ground Truth and Accuracy of Bowl Parameter Estimation

**Figure 7:**
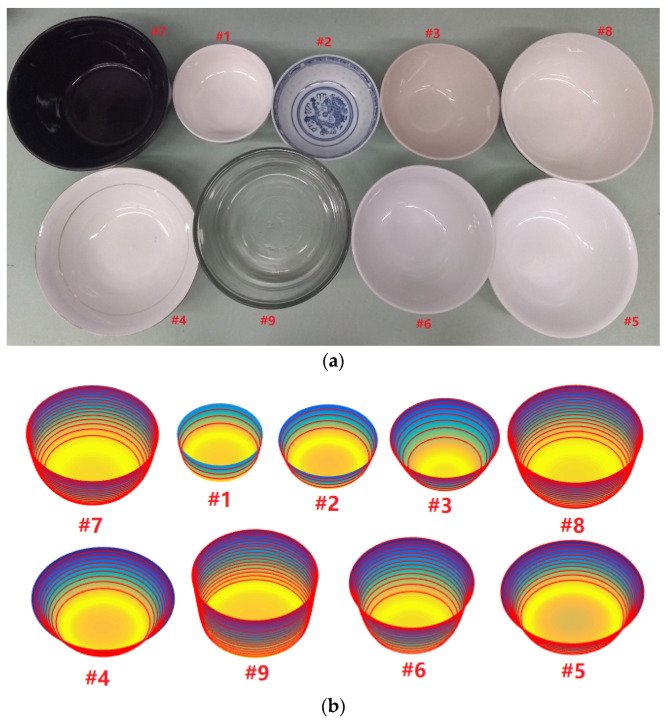
(**a**) Nine bowls used in the experiments and (**b**) reconstructed bowls.

In our experiment, nine commonly used bowls (Figure 7) were investigated. These bowls had different shapes, including the ones with a nearly half-dome shape (#2), steep walls (#1 and #9), and large sizes (#7, #8, and #9). Note that all bowls have circular rims (our algorithm cannot handle bowls with non-circular rims such as those made by hand with irregular shapes). The depths and diameters of these bowls were measured with a ruler. The capacity of each bowl was obtained by pouring water into the bowl carefully until full. The amount of water was measured using a graduated cylinder (Thermo Fisher Scientific, USA). The results were used as the ground truth. Next, an adhesive tape was applied to each bowl and the 3D shape of the bowl was reconstructed using the proposed method. Then, the same set of parameters (depth, diameter, and capacity) were estimated from the reconstructed bowls. Comparisons between the measured and estimated parameters are listed in Table 1. It shows that most relative errors of the estimated bowl volumes are below 5% and the maximum error is 10.6%. We notice that the shape, size, and steepness of the bowl did not significantly affect the reconstruction error. However, if the bowl has a rim curled outward, a larger error could occur (e.g., #5). This was mainly due to the difficulty to define the location of the rim when the rim is widened by the outward curl.

### 3.2. Volume Estimation of Liquid in a Bowl

In this experiment, each bowl was filled with different amounts of red tea. Pictures were then taken using an eButton and a smartphone (Motorola Cruise e5). The eButton is a wearable device worn on the chest and automatically takes a picture every 4 s [62,63]. This device has been used in several dietary studies [3,37,40,64,65,66]. The camera of the eButton has a 170° wide-angle lens. Because the resulting field of view is large, the images obtained contain considerable distortion. To correct the distortion, a series of checkerboard images were taken by the eButton before the study. Then, the MATLAB calibration toolbox [67] was used to pre-process the images. A total of 26 liquid samples in the 9 bowls were tested in this experiment. The actual volumes, measured using a graduated cylinder, were between 90 mL and 550 mL. These volumes were used as ground truth. Each bowl with red tea was photographed by both the smartphone and the eButton.

**Figure 8:**
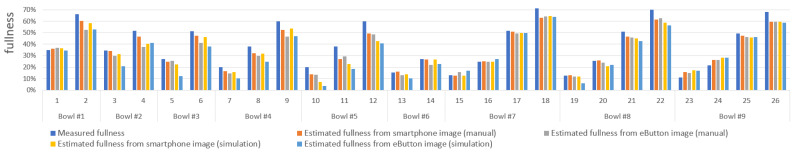
Actual and estimated fullness values from both the smartphone and eButton images using manual estimation and simulation. Each group containing five bars corresponds to one liquid sample. The first bar represents the measured fullness, i.e., the ground truth, and the other four bars correspond to the results of the four estimation methods, respectively.

To facilitate comparisons among samples of different volumes in different bowls, we defined a normalized volumetric measure called “fullness” to represent how full the bowl is with the liquid/food inside. Specifically, the fullness is defined as the ratio (in percentage) of the actual liquid/food volume to the volume of the bowl. Experimentally, we first established the ground truth of fullness for each bowl by calculating the volumetric ratio of the ground truth of the tea volume in each bowl and the volume of the whole bowl. Then, the fullness was estimated using both manual estimation (described in Section 2.2) and simulation methods (described in Section 2.3). Finally, the estimated fullness values were compared with the ground truth value for each sample. Our experimental results of all 26 liquid cases are summarized in Figure 8 and Table 2. It can be observed from Figure 8 that most of the computationally obtained levels of fullness (the last four bars in each group of bars) are less than the ground truth (to be discussed in the Discussion section). For comparison with other published results, we also listed in Table 2 the statistical measures—median value and interquartile range (IQR, the difference between the third and first quartiles)—of the fullness and relative errors of the estimated volume of each sample. The median of the fullness errors over the 26 cases is in the range between −8.7% and −2.8%, and the median of the relative errors is in the range between −18.6% and −7.0%. It can also be observed from Table 2 that the IQR of both the fullness errors and the relative errors are small.

### 3.3. Volume Estimation of Food in a Bowl

In this experiment, the volumes of various amorphous foods were used to evaluate the volume estimation method. This experiment requires an establishment of the ground truth volume. The traditional ground truth is established using the time-consuming water or seed displacement method, which is difficult to implement outside the laboratory. We developed a new method using the measurement of food density. A four-step procedure was performed: (1) A standard measuring cup was filled with a sample of each food taken from the bowl. Then, the net weight of the food within the cup was measured using a digital kitchen weighing scale. The result was denoted by $w_{c}$. (2) The density of the food sample was calculated by $\rho =w_{c}/v_{c}$, where ρ and $v_{c}\;$ represent, respectively, the food density and the volume of the measurement cup (usually 237 mL). (3) The food sample was returned to the bowl and the net weight of the food in the bowl was measured. The result was denoted by $w_{f}$. (4) Finally, the volume of the food in the bowl, $v_{f},\;$ was calculated by $v_{f}=w_{f}/\rho$. We used this value as the ground truth.

**Figure 9:**
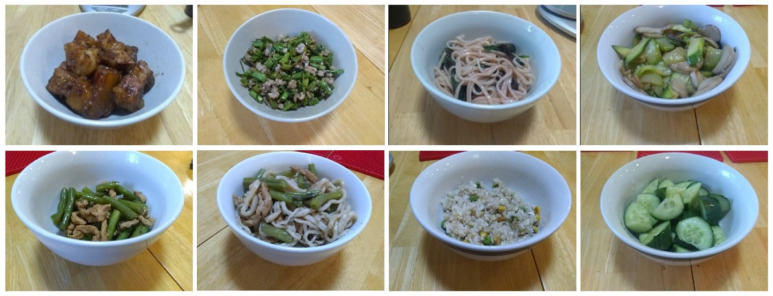
Examples of real food images.

**Figure 10:**
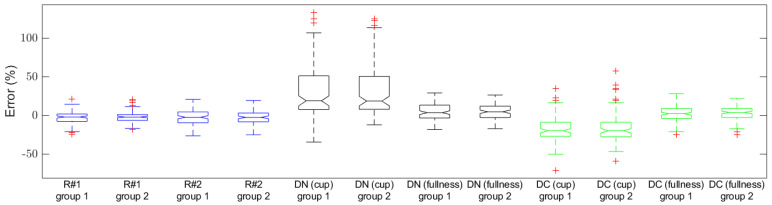
Box plots of estimation errors of fullness estimated by two researchers using our software and by a registered dietitian with prior experience estimating volume from images using direct visualization. R represents the researchers’ estimation, DN represents the dietitian’s estimation with no image cue (i.e., water bottle in the bowl image), and DC represents the dietitian’s estimation with the cue. On each box, the central line represents the median of the errors over all the food samples. The bottom and top edges of the box are, respectively, the first and the third quartiles, which is the IQR. Whiskers are extended to the most extreme data point that is no more than 1.5× IQR from the edge of the box. Points outside the whiskers are plotted individually as pluses, representing potential outliers.

In the volume estimation process, food images were acquired by the same smartphone as the one used in the liquid experiment. Three of the nine bowls shown in Figure 7a (Bowl #3, #6, and #8) were used in this experiment (we made such choices because these bowls were representatives of different sizes). We collected a total of 114 real-world amorphous foods from home kitchens and local restaurants, and 38 different foods were tested for each bowl. Eight example images are shown in Figure 9. Two images were taken for each food with randomly selected distances and viewing angles to investigate the consistency of the proposed method in the variable, but natural, picture-taking environments. These two images were randomly assigned to one of two groups (group 1 or group 2). Next, the food volumes were estimated independently using the method described in Section 2.2 and our computer interface (Figure 5b). To assess inter-rater variability, two researchers participated in the estimation process independently, each researcher estimated food volumes for both groups of the images. Before the estimation started, a training session was given independently to each researcher using six food-containing images. For each image, the researcher estimated the fullness using our software interface. Then, the interface informed the researcher of the ground truth and the estimation error, allowing the researcher to improve his/her performance in consequent estimation sessions. The estimation results of the two researchers are shown in Figure 10 and Table 3. It can be observed that the median values of fullness errors and relative errors of both researchers are less than 5%. We can also see that the results of group 1 and group 2 are similar, indicating the consistency of the proposed approach.

To further compare the performances of our computing tool and direct human estimation (computing tool vs. human), we invited a registered dietitian (with experience in estimating food volumes in images) to participate in this study. The 228 food images were presented to the dietitian in random order on a computer screen. For each image, the dietitian was asked to estimate the food volume in different metrics: one was in measuring cup and the other in fullness. In this study, we tested two types of the information shown on the screen for the dietitian to use as references: one was an image containing the empty bowl together with a set of key measurements of the bowl, including the volume, diameter, and depth, and the other was an image containing the empty bowl and a 500 mL water bottle, in addition to the same set of measurements. The estimation results from the dietitian are also shown in Figure 10 and Table 3. It can be observed that the researchers’ estimates using our computational tool were more accurate than the dietitian’s direct visual estimates. It can also be observed that the accuracies of the two metrics (cup vs. fullness) were markedly different. Although estimated by the same dietitian, the errors using the measuring cup metric were much larger than those using the fullness metric when there was no cue in the image. These estimates improved when a water bottle was presented as a cue in the image. However, they were still not as accurate as the researchers’ estimates using our software.

## 4. Discussion

In this section, we discuss several important issues related to bowl reconstruction and food volume estimation, and ultimately estimation of nutrient intake.

***Assumptions*** Our method for reconstructing the 3D shape of a bowl is based on the following assumptions: (1) the top boundary of the bowl is a perfect circle, (2) the adhesive paper ruler is taped centrally across the bottom and sides of the bowl, (3) the camera lens is located directly above the center of the bowl, and (4) when viewed from the image, the paper ruler is parallel to the bottom edge of the image. However, in practice, bowls, especially those made manually from wood or clay, may not be exactly circular, and the paper ruler may not be taped centrally across the bowl exactly. In addition, the position of the camera is difficult to control precisely when held in one’s hand. Thus, these assumptions are difficult to satisfy exactly. As a result, errors are present in volume estimates. Researchers should be aware of these errors and consider the errors in their implementation of experiments. Here, we note that the last two assumptions could be removed by improving our algorithm. However, a more general form of the mathematical model must be used to replace the simple model shown in Figure 3, but this will cause the bowl reconstruction algorithm to become more complex because it must account for the variations of the camera viewpoint and camera rotation angles. We are exploring mathematical methods to solve this problem.

***Automation*** Although the proposed method is easy to use, automating the reconstruction procedure is still a challenging problem. Currently, both the locations of the ruler markers on the tape and the level of the liquid or food within the bowl must be determined manually from the input image. While the detection of ruler markers can be implemented by specific image processing algorithms, such as segmenting the markers based on color and other features followed by detection of endpoints, the determination of the liquid or food level within the bowl is more challenging, as shown in Figure 9. A “thought process” is required to identify the “peaks” and “valleys” in the free-standing part (i.e., top part) of the food in the bowl. Next, the peaks are imagined to be flattened to fill the valleys. Finally, the imagined leveled surface gives rise to the desired food level. Since the “peaks” and “valleys” are only hinted by the image without specific 3D information, the flattening/filling process is subjective, imprecise, and difficult to implement by the traditional computational means. To meet this challenge, we are developing a machine learning approach trying to solve the food level estimation problem. Although improvements are expected, we may still have to tolerate a certain level of error due to the intrinsic lack of 3D information in the food image.

***Food Volume Metrics*** Our “computing tool vs. human” experiment suggests that, from a food image, direct estimation of volume in a bowl using “cup” as the unit is less precise than estimating the “fullness” of the bowl, even for an experienced registered dietitian. We think it is generally true that, for a human or a computer, the fullness is a better metric than the cup (or any other common volumetric units, e.g., milliliters) for food volume estimation. Although this is still an assertion to be proven rigorously by experiments, we believe that the use of fullness translates the difficult volumetric estimation problem to a much easier size comparison problem between the food and the bowl, since they are both observable from the image. In contrast, the “cup” or “milliliters” are volumetric references outside the image. As additional evidence, we previously investigated the use of a 64-milliliter cube displayed along with the food in the image as an observable volumetric reference which yielded much higher accuracy in food volume estimation than using cups [44].

***Bias in Liquid Volume Estimation*** It has been observed from Figure 8 that the volumetric estimation error for liquid in a bowl was mostly negative. We analyzed all steps in the estimation process and found that the most likely reason was due to the negative error in the liquid level detection step. If the liquid is lightly colored, as in the case of red tea used in our experiment, the detected elliptic boundary between the liquid and the bowl’s interior tends to be smaller than the actual boundary because the liquid near the boundary is shallow, not showing enough color to be detected, either by visual inspection or color-based image segmentation. Attention should be paid to this issue in practice. In addition, the linear relationship between the FAR and food volume is an approximation by observing the simulation results as described in Section 2.3. The inaccuracy of this approximated linear relationship and the inaccuracy of image undistortion may be the reason that causes the biggest estimation error when using the simulation approach for the eButton images.

***Cues and References*** In a recent report, thirty-eight nutritionists, dietitians, and nutrition researchers were invited to estimate portion sizes of two sets of digital food images presenting a meal in a food container (plate or bowl). Even with a standard checkerboard (2D) placed beside the plate or bowl as a cue in every image, the mean percentage difference in portion size was still over 44% and less than one-third of the participants estimated food portion within 10% of the ground truth [36]. A similar conclusion was made in another study [68]. A reason for the relatively poor performance by the dietitian was likely due to the insufficient use of the cues provided. Our experiment indicates that the computer and humans use different cues to estimate food volume from images. While the computer prefers a complete 3D shape model as a volumetric reference, such as the interior surface of the bowl, the human prefers more intuitive cues, such as forks/spoons of known sizes, sizes of certain food components, or even human hands present in the image. Thus, how to maximize the amount of information in the provided cues by a human estimator is an interesting subject to study [68].

## 5. Conclusions

Despite the importance of diet in maintaining human health and preventing chronic diseases, at present, the amount of food still cannot be gauged from images objectively and reliably. One of the challenges is that the bowl as a common food container cannot be measured with acceptable accuracy in the two-dimensional image space. In this work, we have developed a convenient and accurate method to estimate the volumes of both the bowl and the food contained within the bowl from a 2D image. By simply taping a paper ruler centrally across the bottom and sides of the bowl and then taking an image, the size and shape of the bowl are measured computationally. This method can be implemented easily in practice as a pre-procedure before a dietary study. An image processing algorithm is developed to reconstruct the interior surface of the bowl based on the observed distortions of the ruler and ruler markers from the bowl image. With the reconstructed bowl interior, the volumes of amorphous foods are estimated from a quantity called “fullness” defined as the food volume divided by the volume of the bowl. We have compared the performances of human and our computer algorithm using over 200 real-world food samples. The results show that the estimation error by the computer is generally less than the human estimation error, indicating the effectiveness of our approach. The experimental data also indicate that volume estimation using the fullness produces superior results to direct volume estimation. This study has provided a new practical tool for image-based dietary assessment involving bowls as food containers.

## Figures and Tables

**Table 1:** Comparisons of measured (ground truth) and estimated depth, diameter, and capacity of each bowl.

	Bowl #1	Bowl #2	Bowl #3	Bowl #4	Bowl #5	Bowl #6	Bowl #7	Bowl #8	Bowl #9
Actual diameter (mm)	101	110	121	157	153	135	151	152	143
Calculated diameter(mm)	96.9	110.6	118.7	154.6	154.3	135.2	142.7	145.4	135.6
Relative error * (%)	−4.1	0.5	−1.9	−1.5	0.8	0.1	−5.5	−4.3	−5.2
Actual depth (mm)	45	42	52	44	43	70	59	61	65
Calculated depth (mm)	47.3	39.1	52.5	42.2	42.9	67.9	62.3	64.1	68.7
Relative error * (%)	5.1	−6.9	1	−4.1	−0.2	−3	5.6	5.1	5.7
Actual capacity (mL)	288	291	371	500	500	642	773	787	810
Calculated capacity (mL)	288.7	288	362.9	496.9	553	661.4	769.4	803.4	851.6
Relative error * (%)	0.2	−1	−2.2	−0.6	10.6	3	−0.5	2.1	5.1

* Relative error = (measured value − actual value)/actual value × 100%.

**Table 2:** Estimation errors using smartphone and eButton images.

		Smartphone Images		eButton Images	
		Manual	Simulation	Manual	Simulation
Fullness	Mean ± s.t.d.	−3.2% ± 4.3%	−4.8% ± 5.7%	−4.9% ± 5.2%	−7.7% ± 7.3%
	Root mean square error (RMS)	5.3%	7.4%	7.1%	10.6%
	Median	−2.8%	−4.4%	−5.1%	−8.7%
	IQR	6.7%	7.0%	7.0%	10.0%
Relative error *	Median	−7.0%	−9.9%	−9.5%	−18.6%
	IQR	12.8%	13.4%	16.0%	31.3%

* Relative error = (measured value − actual value)/actual value × 100%.

**Table 3:** Errors of the estimated volumes of the 228 foods.

		Fullness		Relative Error *	
		Median	IQR	Median	IQR
R#1 (Researcher #1)	Group 1	−1.6%	9.4%	−3.3%	21.8%
	Group 2	−1.7%	7.5%	−3.7%	15.5%
R#2 (Researcher #2)	Group 1	−2.0%	14.0%	−2.6%	25.4%
	Group 2	−2.1%	11.4%	−3.3%	22.2%
DN (cup) (Dietitian: cup estimation, no cue)	Group 1	19.3%	43.7%	50.0%	79.4%
	Group 2	19.0%	42.7%	50.0%	75.0%
DN (fullness) (Dietitian: fullness estimation, no cue)	Group 1	4.0%	16.7%	10.2%	38.9%
	Group 2	5.1%	15.0%	9.7%	35.4%
DC (cup) (Dietitian: cup estimation, with the cue)	Group 1	−19.4%	18.2%	−43.6%	27.2%
	Group 2	−19.4%	18.7%	−47.4%	24.6%
DC (fullness) (Dietitian: fullness estimation, with the cue)	Group 1	2.6%	12.9%	9.2%	32.0%
	Group 2	4.2%	11.8%	8.4%	34.8%

* Relative error = (measured value − actual value)/actual value × 100%.

## Data Availability

The datasets used and/or analyzed during the current study are available from the corresponding author upon request.
